# Gap-PCR Screening for Common Large Deletional Mutations of β-Globin Gene Cluster Revealed a Higher Prevalence of the Turkish Inversion/Deletion (δβ)0 Mutation in Antalya

**DOI:** 10.4274/tjh.2014.0242

**Published:** 2016-05-16

**Authors:** Türker Bilgen, Özden Altıok Clark, Zeynep Öztürk, M. Akif Yeşilipek, İbrahim Keser

**Affiliations:** 1 Akdeniz University Faculty of Medicine, Department of Medical Biology and Genetics, Antalya, Turkey; 2 Namık Kemal University Central Research Laboratory (NABİLTEM), Tekirdağ, Turkey; 3 Akdeniz University Faculty of Medicine, Department of Medical Genetics, Antalya, Turkey; 4 Akdeniz University Faculty of Medicine, Department of Pediatric Hematology and Oncology, Antalya, Turkey

**Keywords:** Deletional mutations, Turkish inversion/deletion (δβ)0 mutation, Gap-PCR, β-Globin gene cluster

## Abstract

**Objective:**

Although the calculated carrier frequency for point mutations of the β-globin gene is around 10% for Antalya Province, nothing is known about the profile of large deletional mutations involving the β-globin gene. In this study, we aimed to screen common deletional mutations in the β-globin gene cluster in patients for whom direct DNA sequencing was not able to demonstrate the mutation(s) responsible for the disease phenotype.

**Materials and Methods:**

Thirty-one index cases selected with a series of selection events among 60 cases without detected β-globin gene mutation from 580 thalassemia-related cases tested by direct sequencing over the last 4 years in our diagnostic center were screened for the most common 8 different large deletional mutations of the β-globin gene cluster by gap-PCR.

**Results:**

We detected 1 homozygous and 9 heterozygous novel unrelated cases for the Turkish inversion/deletion (δβ)0 mutation in our series of 31 cases. Our study showed that the Turkish inversion/deletion (δβ)0 mutation per se accounts for 16.6% of the unidentified causative alleles and also accounts for 1.5% of all detected mutations over the last 4 years in our laboratory.

**Conclusion:**

Since molecular diagnosis of deletional mutations in the β-globin gene cluster warrants different approaches, it deserves special attention in order to provide prenatal diagnosis and prevention opportunities to the families involved. We conclude that the Turkish inversion/deletion (δβ)0, as the most prevalent deletional mutation detected so far, has to be routinely tested for in Antalya, and the gap-PCR approach has valuable diagnostic potential in the patients at risk.

## Abstract (tr)

**Amaç:**

Beta-globin genindeki nokta mutasyonlarının sıklığı Antalya bölgesi için yaklaşık %10 olarak belirlenmiş olmasına rağmen, beta-globin genini içine alan büyük delesyonel tip mutasyonların profili hakkında hiçbir şey bilinmemektedir. Bu çalışmada, DNA dizi analizi yöntemiyle beta-globin geninde hastalığın oluşmasından sorumlu mutasyon(lar) tespit edilememiş talasemili olgularda beta-globin gen kümesinde yaygın görülen büyük delesyonel mutasyonları taramayı amaçladık.

**Gereç ve Yöntemler:**

Son dört yıl boyunca tanı merkezimizde DNA dizi analizi yöntemiyle test edilmiş, talasemiyle ilişkilendirilen 580 olgu arasından öncelikle beta-globin geni mutasyonu belirlenememiş 60 olgu seçildi. Bu 60 olgu arasından bir seri seleksiyon işlemi uygulanarak nihai olarak belirlenmiş 31 hasta, beta-globin gen kümesinde en yaygın görülen sekiz farklı büyük delesyonel tip mutasyon için gap-PCR yöntemiyle tarandı.

**Bulgular:**

Otuz bir olgudan oluşan serimiz içerisinde, Türk tipi inversiyon/delesyon (δβ)0 mutasyonu açısından heterozigot olan dokuz yeni olgu ve homozigot olan bir yeni olgu belirlendi. Çalışmamız Türk tipi inversiyon/delesyon (δβ)0 mutasyonunun, laboratuvarımızda son dört yıl boyunca tespit edilmiş tüm mutasyonların %1,5’ini ve DNA dizi analizi yöntemiyle mutasyon tespit edilemeyen alellerin ise %16,6’sını oluşturduğunu gösterdi.

**Sonuç:**

Beta-globin gen kümesinde delesyonel tip mutasyonlar farklı moleküler yöntemlerle tespit edilebilir. Bu durum prenatal teşhis ve hastalığı önleme fırsatı sağlayabildiği için özel bir ilgi gerektirmektedir. Sonuç olarak, toplumumuzda şu ana kadar belirlenmiş en sık görülen delesyonel tip mutasyon olan Türk tipi inversiyon/delesyon (δβ)0 mutasyonu Antalya’da rutin olarak test edilmelidir ve gap-PCR yöntemi risk altındaki hastalar için önemli bir tanı potansiyeline sahiptir.

## INTRODUCTION

Beta-thalassemia (β-thal) is generally caused by point mutations in the β-globin gene. However, there are at least 80 different large deletional mutations in the β-globin gene cluster described in the human hemoglobin variant (HbVar) database. While only the β-globin gene is partially or completely removed in some of those deletions, the δ-globin gene or δ- and γ-globin genes are deleted in addition to the β-globin gene in some others [1,2]. It was also stated that 10% of the β-globin gene mutations are large deletions causing phenotypes associated with β-thal [3]. The phenotypes produced by deletions in the β-globin gene cluster are classified according to the gene(s) involved, such as β-thal, δβ-thal, εγδβ-thal, and hereditary persistence of fetal hemoglobin (HPFH) [4]. Despite general carrier frequency for β-globin gene mutations being reported at 2% for Turkey and at as high as 10% for Antalya Province, large deletional mutations in the β-globin gene cluster have rarely been reported so far and there is no systemic study on mutation profiles of large deletional mutations in the β-globin gene cluster in Turkey [4,5,6,7,8,9]. On the other hand, the number of studies on variety and allelic frequencies of large deletions in the β-globin gene cluster has been growing recently [2,3,10,11,12]. Previous studies revealed that HPFH-1, HPFH-2, HPFH-3, Sicilian (δβ)0-thal, Chinese Gγ(Aγδβ)0-thal, Hb Lepore, Asian-Indian inversion-deletion Gγ(Aγδβ)0-thal, and Turkish inversion-deletion (δβ)0-thal mutations are among the most recurrent large deletional mutations in the β-globin gene cluster [10,13].

Detection of large deletions of the β-globin gene cluster has recently become an important issue because of its significance in evaluation of unresolved thalassemia-related cases and in disease prevention. On the other hand, commonly used diagnostic tests targeting point mutations and small insertions-deletions of the β-globin gene are not suitable for detection of large deletional mutations. Therefore, molecular detection of large deletions needs different approaches in the laboratory. Researchers have recently applied strategies like Southern blotting, FISH, quantitative polymerase chain reaction (PCR), multiplex ligation-dependent probe amplification (MLPA), and gap-PCR for molecular detection of large deletional mutations of the β-globin gene cluster [10,12,14,15,16]. Among them, gap-PCR is a fast and reliable method allowing us to detect the previously characterized mutations [3,13]. In this study, we screened patients in whom we were not able to find the underlying β-globin gene mutation(s) by direct DNA sequencing for the 8 different common deletional mutations of the β-globin gene cluster by gap-PCR.

## MATERIALS AND METHODS

### Patients

Among the 580 patients who were tested in our diagnostic laboratory for β-globin gene mutations by direct DNA sequencing between July 2008 and July 2012, a total of 60 unrelated patients who had either no causative β-globin gene mutation(s) by sequencing or no detectable PCR amplification for the β-globin gene were initially selected. Being homozygous for all common intragenic single-nucleotide polymorphisms detected by sequence analyses was then used as the second inclusion criterion for its potential to indicate hemizygosity. Finally, the 31 most probable candidates were screened by gap-PCR for the 8 different known deletions of the β-globin gene cluster. Out of these 31 patients included in the study, 21 had a mild phenotype without any β-globin gene mutation, while the remaining 10 were moderately to seriously affected by the disease with either one or no detected causative mutations.

All hematological and clinical findings were collected with the informed consent of the patients. Hematological indices were obtained with an automated cell counter (Abbott Cell DYN3700; Abbott Laboratories, Abbott Park, IL, USA). The HbA2 and HbF levels were measured by high-performance liquid chromatography (VARIANT; Bio-Rad Laboratories, Hercules, CA, USA).

### Sequence Analyses and Gap-PCR Screening for the 8 Known Deletional Mutations of the β-Globin Gene

Following the isolation of genomic DNA with a commercial kit (AxyPrep Blood Genomic DNA Miniprep Kit; Axygen Biosciences Inc., Union City, CA, USA), the β-globin gene was amplified as 2 PCR fragments (from the -101 position to the Poly-A signal) using 30-50 ng of genomic DNA in 25-µL reaction volumes. The PCR mixture contained 12.5 µL of 2X PCR master mix and 5 pmol of each primer (GML, Wollerau, Switzerland). The sequencing was performed using the BigDye Terminator v3.1 Cycle Sequencing Kit and an ABI Prism 3130 Genetic Analyzer (Applied Biosystems, Foster City, CA, USA).

The deletional mutations were chosen by taking into account ethnic background and according to the published frequencies [10]. Gap-PCR protocols and the primers for the deletional mutations HPFH-1, HPFH-2, HPFH-3, Sicilian (δβ)0-thal, Chinese Gγ(Aγδβ)0-thal, Hb Lepore, Asian-Indian inversion-deletion Gγ(Aγδβ)0-thal, and Turkish inversion-deletion (δβ)0-thal were used as previously described elsewhere [13].

## RESULTS

Among the 8 different known deletions of the β-globin gene cluster mentioned above, only the Turkish inversion-deletion (δβ)0 mutation was detected in 10 patients in our series. We found that 9 were heterozygous and 1 was homozygous for the Turkish inversion-deletion (δβ)0 mutation. The hematological indices and molecular findings of 7 heterozygous patients and 1 homozygous patient are summarized in Table 1. Hematological indices were not available for 2 heterozygous patients, males of 24 and 16 years old. Sequence analyses of mutation-related gap-PCR bands of 10 patients showed that there was no variation in sequence or at breakpoints of the Turkish inversion-deletion (δβ)0 mutation. Sequence analyses determined that the exact breakpoints positions were 5.255,764 and 5.244,281 for upstream deletion (11,484 bp) and 5.236,654 and 5.235,062 (1592 bp) for downstream deletion according to NCBI reference sequence NC_000011.9, chromosome 11 GRCh37.p13 primary assembly.

## DISCUSSION

**Figure 1:**
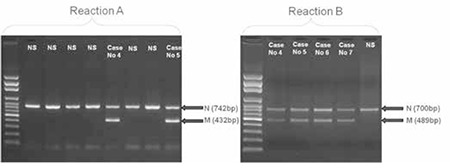
Representative samples of Turkish-type inversion/deletion (δβ)0 mutation detected by gap-PCR. For reaction A testing the upstream breakage of the mutation, the upper band (742 bp) corresponds to normal results and the lower band (432 bp) to the mutation. Case 4 and Case 5 are heterozygous as both have normal and mutation-related polymerase chain reaction fragments. For reaction B testing the downstream breakage of the mutation, the upper band (700 bp) corresponds to normal results and the lower band (489 bp) to the mutation. Cases 4, 5, 6, and 7 show both normal and mutation-related polymerase chain reaction fragments, confirming that they are heterozygous for the mutation. NS: Normal sample, N: normal, M: mutation, 252x91 mm (72x72 dpi).

While nearly 25 different β-globin gene mutations have been reported for Antalya Province as well as for Turkey so far, the large deletional-type mutations of the β-globin gene cluster have not been systematically investigated [4,6,17]. It has been suggested that 10% of the causative alleles cannot be easily detected by routine methods in β-thal-associated phenotypes [18]. This proportion in our survey was similar to the literature. Large deletional mutations might be somewhat responsible for this challenge. In this regard, the Turkish inversion/deletion (δβ)0 mutation per se accounts for approximately 16.6% of the unidentified causative alleles and accounts for 1.5% of all detected mutations from the last 4 years in our laboratory. Among the 10 new unrelated cases of the Turkish inversion-deletion (δβ)0 mutation detected in this study, only one seemed to be homozygous. The gap-PCR technique is able to reliably detect the heterozygous state of this type of mutation by showing both normal and mutation-related bands on agarose gel (Figure 1). On the other hand, this technique does not exclude the possibility of the presence of another larger deletional mutation such as the second mutation in the patient found as homozygous in our study. This should be considered as a technical limitation of gap-PCR; in addition, its usage is limited to known deletional mutations. Gap-PCR analyses of the parents of the patient would help to clarify such a situation; however, we were not able to perform this analysis in this family.

It has been demonstrated that the deletions in the β-globin gene cluster may cause HPFH, which is characterized by high HbF levels reducing the disease severity [19]. While the patients with deletions including the δ- and β-globin genes tend to have mild phenotypes, patients with larger deletions involving γ-globin genes have severe clinical phenotypes because of the lack of the compensatory effect of fetal Hb [20]. Furthermore, recent studies on hemoglobin switching events have revealed that there is a binding site between the δ- and γ-globin genes for BCL11, which is a repressor of γ-globin genes. The deletion of this cis-acting element seems to be related to higher HbF levels [9]. The Turkish type of inv/del (δβ)0 thalassemia was first characterized at the molecular level by Kulozik et al. in a Turkish patient living in Germany with normal HbA2 and elevated HbF levels in 1992 [21]. It was also associated with elevated HbF and normal HbA2 levels in another later study [22]. Our study revealed that 7 out of 9 patients carrying the Turkish inv/del (δβ)0 had elevated HbF levels, while the remaining 2 had normal HbF levels. This controversial observation can be explained by other factors that may modify the hematological expression of this mutation. Such a situation was reported in δβ-thalassemia before by Öner et al. [23]. This phenomenon shows that a small proportion of the carriers of the Turkish inv/del (δβ)0 mutation may not have elevated HbF levels, which should be considered in case selection for mutation screening.

Another important point is that the molecular detection of large deletional mutations in the β-globin gene cluster is extremely important for families at risk and seeking prevention. Because their detection requires special attention, this type of mutation may sometimes compromise the prenatal diagnosis in laboratories used to focusing on point mutations and small ins/del-type mutations of the β-globin gene. Despite not being useful for previously uncharacterized deletions, gap-PCR is the easiest and most precise way of detecting previously characterized recurrent deletions. For these reasons, and taking into account the relatively higher incidence of the Turkish-type inv/del (δβ)0 mutation in Antalya Province, we suggest that it is worthwhile to screen for this mutation in Turkish patients when the first-line diagnostic tests such as sequencing and strip assay fail to detect the causative mutation(s).

Gap-PCR is the cheapest and fastest method for the detection of large deletional mutations. Nevertheless, the approach has specific requirements for being used as a diagnostic tool, such as positive controls, and the targeted mutation has to be previously well described. Having positive controls is important for optimization and validation of gap-PCR. Without well-optimized protocols, gap-PCR should not be used as a routine diagnostic method. In addition to the possibility of false negativity, positive results should also be confirmed by family study when the parents are available. We had positive controls for the Turkish-type inv/del (δβ)0 mutation prior to this study, but not for the other types of mutations that we screened. This could be considered as a limitation of our study. The other patients in whom we could detect none of the deletions screened in our study are strong candidates for screening for either other previously described but rarer or completely novel deletional mutations. Therefore, there is need for further analyses in order to resolve these cases. MLPA and array comparative genomic hybridization methods are strong tools to investigate possible novel and rare deletional mutations. MLPA is currently the more commonly used approach for detection of large deletions affecting a particular region of the genome, but its coverage is limited to the probe set designed. We are planning a MLPA study for the patients who had no positive findings in our gap-PCR screening. On the other hand, not only the patients whose mutation(s) were not identified but also even homozygous patients for one particular parental β-globin gene mutation detected by sequencing or strip assay should be investigated for deletional mutations in order to find out the exact second hit leading to thalassemia intermedia or major phenotypes.

## Ethics

Ethics Committee Approval: Retrospective study, Informed Consent: It was taken.

## Figures and Tables

**Table 1:**
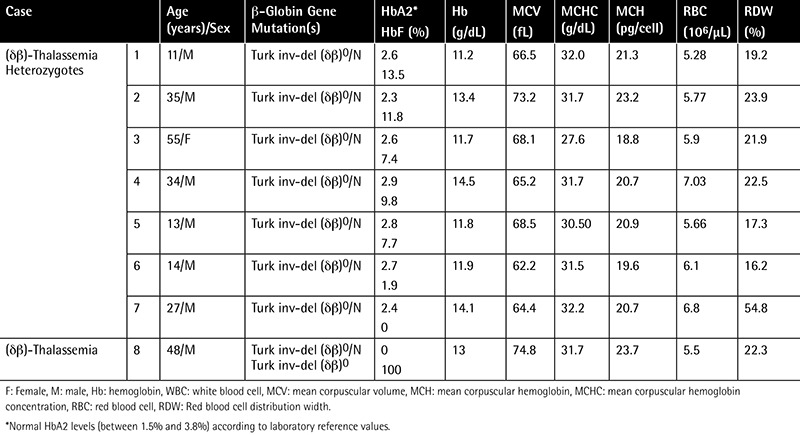
Hematological findings of the patients with Turkish inversion-deletion (δβ)0 mutation.
